# Supplementary material for: What Is on the Menu?—A Quantitative Analysis on Label Format among (Potential) Restaurant Guests and Restaurant Owners
Source: Int J Environ Res Public Health. 2021 Nov 27;18(23):12500. doi: 10.3390/ijerph182312500 (PMC8656631; doi:10.3390/ijerph182312500)
Supplement: Supplementary file 1 [file ijerph-18-12500-s001.zip › Supplementary S1_Questionnaires.pdf]

Supplementary S1. Questionnaires

A. Questionnaire restaurant guests

	**Question**
1.	What is your gender?<br>o Male<br>o Female
2.	What is your age?<br>____________________
3.	What is your residence?<br>________________________________
4.	I am:<br>o High school student<br>o College student<br>o Working, full time (36 or more hours per week)<br>o Working, parttime (less than 36 hours per week)<br>o (currently unemployed<br>o Stay-at-home as a mom or dad<br>o Incapacitated<br>o Retired
5.	What is your monthly income (net per month)?<br>o Less than € 1500<br>o €1500 - €2000<br>o €2000 - €3000<br>o €3000 - €4000<br>o €4000 - €5000<br>o €5000 - €6000<br>o €6000 - €7000<br>o €7000 - €10 000<br>o More than €10 000
6.	What is your highest degree or level of education?<br>(graduated or studying at the moment)<br>o Primary school<br>o Lower vocational education<br>o Preparatory secondary vocational education<br>o Sr. Secondary vocational education<br>o Sr. General secondary & pre-university education<br>o Higher professional education<br>o Academic higher education
7.	On average, how often do you dine out in a restaurant (**not** fast food)?<br>o Daily (every day)<br>o Weekly (1-2 times a week)<br>o Monthly (1-2 times a month)

	o Less than once a month
8.	When dining out, this is in a:<br>o Business setting<br>o Social setting<br>o Both business and social setting
9.	For the questions below, indicate whether you agree or not (1=disgree, 5=agree)<br><br>Living life in best possible health is very important to me.<br>☐ 1 ☐ 2 ☐ 3 ☐ 4 ☐ 5<br><br>Eating right, exercising and relax timely will keep me healthy for life.<br>☐ 1 ☐ 2 ☐ 3 ☐ 4 ☐ 5<br><br>My health depends on how well I take care of myself.<br>☐ 1 ☐ 2 ☐ 3 ☐ 4 ☐ 5<br><br>I actively try to prevent diseases and illness.<br>☐ 1 ☐ 2 ☐ 3 ☐ 4 ☐ 5<br><br>I do everything I can to stay healthy.<br>☐ 1 ☐ 2 ☐ 3 ☐ 4 ☐ 5
10.	What do you consider important when choosing a dish in a restaurant<br>(1 = important, 5 = very important)<br><br>Taste<br>☐ 1 ☐ 2 ☐ 3 ☐ 4 ☐ 5<br><br>Price<br>☐ 1 ☐ 2 ☐ 3 ☐ 4 ☐ 5<br><br>Health aspects<br>☐ 1 ☐ 2 ☐ 3 ☐ 4 ☐ 5<br><br>Chefs' recommendations<br>☐ 1 ☐ 2 ☐ 3 ☐ 4 ☐ 5
11.	To what extent would you let a menu label influence your menu choice?<br>(1= not, 5 =a lot)<br>☐ 1 ☐ 2 ☐ 3 ☐ 4 ☐ 5
12.	When a dish contains a label to indicate the 'healthy choice', I expect this dish to be 1= not tasty, 5 = very tasty.<br>☐ 1 ☐ 2 ☐ 3 ☐ 4 ☐ 5
13.	Would you like to know the nutritional value of your restaurant dish?

- Yes
- No

Menu labeling is the labeling of dishes on the menu that meet certain health criteria. This way guests can see which dishes are a conscious choice. Menu labeling is already widely used at fast food chains such as McDonalds and Burger King, but rarely in the traditional restaurants and catering.

14. In which setting would you be interested in menu labeling?

- In a social setting
- In a business setting
- In both
- I am not intested

Below some questions will follow with regard to three developed menu labels.
The next three questions relate to the image below. Please take a proper look at this image.

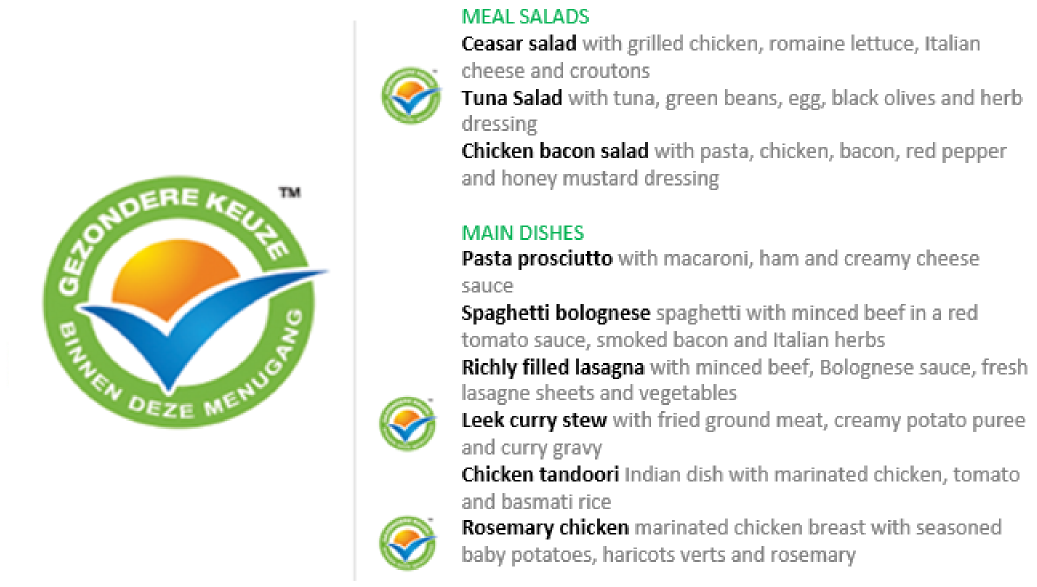

15. What does this menu labeling format communicates according to you?
(1 = not, 5 = strongly)

Good to prevent heart disease	1	2	3	4	5
Good nutrient composition	1	2	3	4	5
Low energy	1	2	3	4	5
Low in fat	1	2	3	4	5
Conscious choice	1	2	3	4	5
Organic	1	2	3	4	5
Sustainable	1	2	3	4	5
Healthy choice	1	2	3	4	5
Tasteful	1	2	3	4	5

16	To what extent would this menu label format influence your menu choice from a restaurant menu?<br>(1= not, 5 =a lot)<br><br>☐ 1 ☐ 2 ☐ 3 ☐ 4 ☐ 5
17	To what extent do you expect dishes with this menu label format to actually be a healthy choice.<br>(1 = not, 5 = very strongly)<br><br>☐ 1 ☐ 2 ☐ 3 ☐ 4 ☐ 5

The next three questions relate to the image below. Please take a proper look at this image.

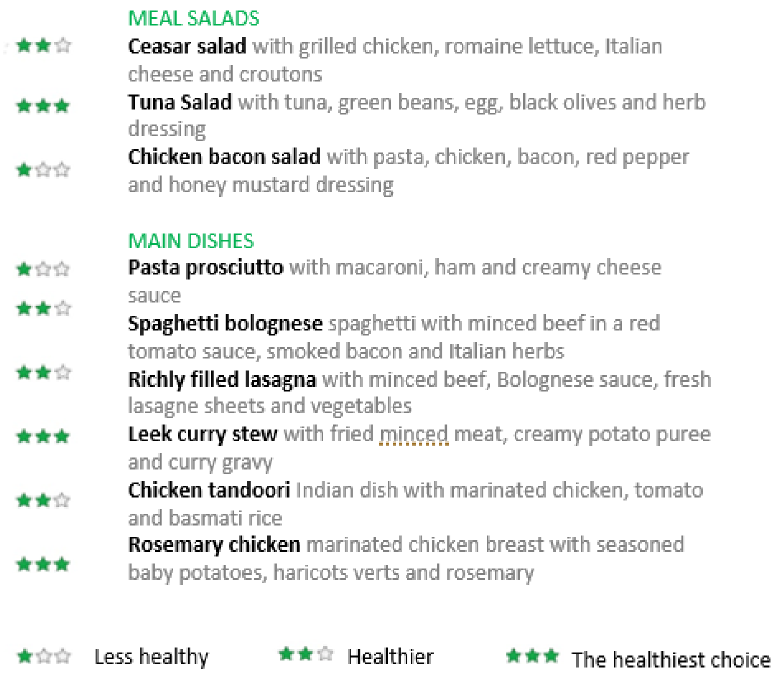
MEAL SALADS

★★☆ **Ceasar salad** with grilled chicken, romaine lettuce, Italian cheese and croutons

★★★ **Tuna Salad** with tuna, green beans, egg, black olives and herb dressing

★☆☆ **Chicken bacon salad** with pasta, chicken, bacon, red pepper and honey mustard dressing

MAIN DISHES

★☆☆ **Pasta prosciutto** with macaroni, ham and creamy cheese sauce

★★☆ **Spaghetti bolognese** spaghetti with minced beef in a red tomato sauce, smoked bacon and Italian herbs

★★☆ **Richly filled lasagna** with minced beef, Bolognese sauce, fresh lasagne sheets and vegetables

★★★ **Leek curry stew** with fried minced meat, creamy potato puree and curry gravy

★★☆ **Chicken tandoori** Indian dish with marinated chicken, tomato and basmati rice

★★★ **Rosemary chicken** marinated chicken breast with seasoned baby potatoes, haricots verts and rosemary

★☆☆ Less healthy ★★☆ Healthier ★★★ The healthiest choice

18 What does this menu labeling format communicates according to you?
(1 = not, 5 = strongly)

Good to prevent heart disease	1	2	3	4	5
Good nutrient composition	1	2	3	4	5
Low energy	1	2	3	4	5
Low in fat	1	2	3	4	5
Conscious choice	1	2	3	4	5
Organic	1	2	3	4	5
Sustainable	1	2	3	4	5
Healthy choice	1	2	3	4	5
Tasteful	1	2	3	4	5

19	To what extent would this menu label format influence your menu choice from a restaurant menu?<br>(1= not, 5 =a lot)<br><br>☐ 1 ☐ 2 ☐ 3 ☐ 4 ☐ 5
20	To what extent do you expect dishes with this menu label format to actually be a healthy choice.<br>(1 = not, 5 = very strongly)<br><br>☐ 1 ☐ 2 ☐ 3 ☐ 4 ☐ 5

The next three questions relate to the image below. Please take a proper look at this image.

Kcal	MEAL SALADS
465	**Ceasar salad** with grilled chicken, romaine lettuce, Italian cheese and croutons
460	**Tuna Salad** with tuna, green beans, egg, black olives and herb dressing
740	**Chicken bacon salad** with pasta, chicken, bacon, red pepper and honey mustard dressing
	MAIN DISHES
690	**Pasta prosciutto** with macaroni, ham and creamy cheese sauce
580	**Spaghetti bolognese** spaghetti with minced beef in a red tomato sauce, smoked bacon and Italian herbs
530	**Richly filled lasagna** with minced beef, Bolognese sauce, fresh lasagne sheets and vegetables
585	**Leek curry stew** with fried ground meat, creamy potato puree and curry gravy
590	**Chicken tandoori** Indian dish with marinated chicken, tomato and basmati rice
490	**Rosemary chicken** marinated chicken breast with seasoned baby potatoes, haricots verts and rosemary

21. What does this menu labeling format communicates according to you?
(1 = not, 5 = strongly)

Good to prevent heart disease	1	2	3	4	5
Good nutrient composition	1	2	3	4	5
Low energy	1	2	3	4	5
Low in fat	1	2	3	4	5
Conscious choice	1	2	3	4	5
Organic	1	2	3	4	5
Sustainable	1	2	3	4	5
Healthy choice	1	2	3	4	5
Tasteful	1	2	3	4	5

22. To what extent would this menu label format influence your menu choice from a restaurant menu?
(1= not, 5 =a lot)

☐ 1 ☐ 2 ☐ 3 ☐ 4 ☐ 5

23.	To what extent do you expect dishes with this menu label format to actually be a healthy choice.<br>(1 = not, 5 = very strongly)<br><br>☐ 1 ☐ 2 ☐ 3 ☐ 4 ☐ 5
24.	When a * is displayed on a restaurant menu, do you search on the menu for its meaning (1= never, 5 = always)?<br>☐ 1 ☐ 2 ☐ 3 ☐ 4 ☐ 5
25.	Which menu label format to communicate that a dish is a healthy and conscious choice do you prefer most?<br>o Logo (menu label 1)<br>o Ranking (menu label 2)<br>o Information (menu label 3)

B. Questionnaire restaurant owners

1.	What is your gender?<br>o Male<br>o Female
2.	What is your age?<br>____________________
3.	What is your highest degree or level of education?<br>(graduated or studying at the moment)<br>o Primary school<br>o Lower vocational education<br>o Preparatory secondary vocational education<br>o Sr. Secondary vocational education<br>o Sr. General secondary & pre-university education<br>o Higher professional education<br>o Academic higher education
Menu labeling is the labeling of dishes on the menu that meet certain health criteria. This way guests can see which dishes are a conscious choice. Menu labeling is already widely used at fast food chains such as McDonalds and Burger King, but rarely in the traditional restaurants and catering.	
4.	Do you think it would be possible to implement menu labeling in restaurants?<br>(1 = easy, 5 = difficult)<br><br>☐ 1 ☐ 2 ☐ 3 ☐ 4 ☐ 5
5.	In what areas do you anticipate problems when implementing menu labeling?

(1 = many problems, 5 = no problems)

	1	2	3	4	5
Explaining the meaning of the menu label to (potential) restaurant guests					
Coming up with dishes that meet the criteria for the menu label					
Preparing dishes that meet the criteria for the menu label					
Time it takes to calculate the nutritional information of the dishes					

6. How much value do you think your guests attach to health aspects of the menu items when dining out?
(1 = no value, 5 = a lot of value)

☐ 1 ☐ 2 ☐ 3 ☐ 4 ☐ 5

7. How much value do you attach yourself to preparing healthy dishes for you restaurant guests?
(1 = no value, 5 = a lot of value)

☐ 1 ☐ 2 ☐ 3 ☐ 4 ☐ 5

Below some questions will follow with regard to three developed menu labels.
The next three questions relate to the image below. Please take a proper look at this image.

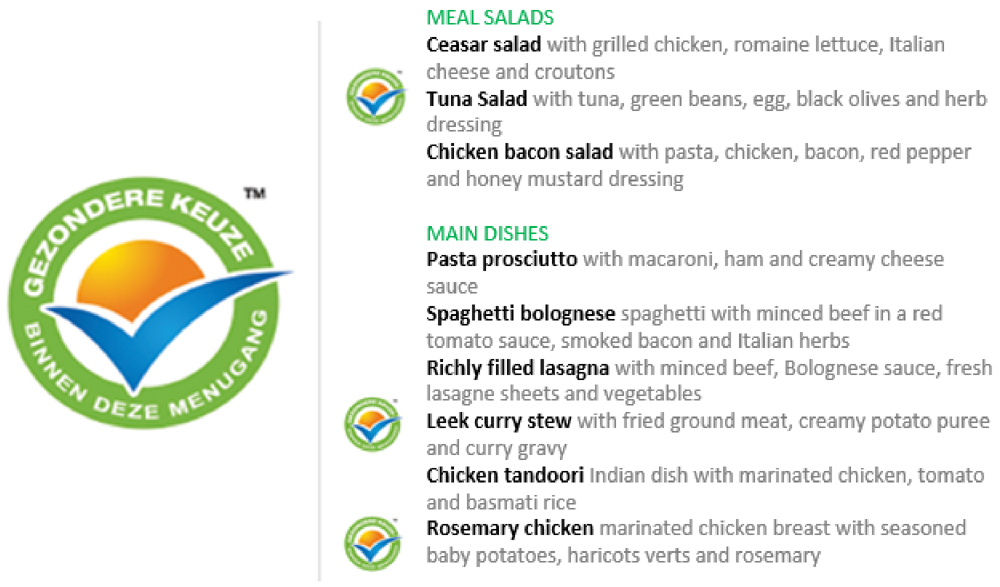

8. What does this menu labeling format communicates according to you?
(1 = not, 5 = strongly)

Good to prevent heart disease	1	2	3	4	5
Good nutrient composition	1	2	3	4	5
Low energy	1	2	3	4	5
Low in fat	1	2	3	4	5
Conscious choice	1	2	3	4	5
Organic	1	2	3	4	5
Sustainable	1	2	3	4	5
Healthy choice	1	2	3	4	5
Tasteful	1	2	3	4	5

9. To what extent would this menu label format influence restaurant guests' menu choice according to you?
(1= not, 5 =a lot)

☐ 1 ☐ 2 ☐ 3 ☐ 4 ☐ 5

The next three questions relate to the image below. Please take a proper look at this image.

MEAL SALADS

★★☆ **Ceasar salad** with grilled chicken, romaine lettuce, Italian cheese and croutons

★★★ **Tuna Salad** with tuna, green beans, egg, black olives and herb dressing

★☆☆ **Chicken bacon salad** with pasta, chicken, bacon, red pepper and honey mustard dressing

MAIN DISHES

★☆☆ **Pasta prosciutto** with macaroni, ham and creamy cheese sauce

★★☆ **Spaghetti bolognese** spaghetti with minced beef in a red tomato sauce, smoked bacon and Italian herbs

★★☆ **Richly filled lasagna** with minced beef, Bolognese sauce, fresh lasagne sheets and vegetables

★★★ **Leek curry stew** with fried minced meat, creamy potato puree and curry gravy

★★☆ **Chicken tandoori** Indian dish with marinated chicken, tomato and basmati rice

★★★ **Rosemary chicken** marinated chicken breast with seasoned baby potatoes, haricots verts and rosemary

★☆☆ Less healthy ★★☆ Healthier ★★★ The healthiest choice

10. What does this menu labeling format communicates according to you?
(1 = not, 5 = strongly)

Good to prevent heart disease	1	2	3	4	5
Good nutrient composition	1	2	3	4	5
Low energy	1	2	3	4	5
Low in fat	1	2	3	4	5
Conscious choice	1	2	3	4	5
Organic	1	2	3	4	5

Sustainable	1	2	3	4	5
Healthy choice	1	2	3	4	5
Tasteful	1	2	3	4	5

11. To what extent would this menu label format influence restaurant guests' menu choice according to you?
(1= not, 5 =a lot)

☐ 1 ☐ 2 ☐ 3 ☐ 4 ☐ 5

The next three questions relate to the image below. Please take a proper look at this image This menu indicates how much energy (kcal) a dish contains. Recommended per day: women 2000 kcal, men: 2500 kcal.

Kcal	MEAL SALADS
**465**	**Ceasar salad** with grilled chicken, romaine lettuce, Italian cheese and croutons
**460**	**Tuna Salad** with tuna, green beans, egg, black olives and herb dressing
**740**	**Chicken bacon salad** with pasta, chicken, bacon, red pepper and honey mustard dressing
	MAIN DISHES
**690**	**Pasta prosciutto** with macaroni, ham and creamy cheese sauce
**580**	**Spaghetti bolognese** spaghetti with minced beef in a red tomato sauce, smoked bacon and Italian herbs
**530**	**Richly filled lasagna** with minced beef, Bolognese sauce, fresh lasagne sheets and vegetables
**585**	**Leek curry stew** with fried ground meat, creamy potato puree and curry gravy
**590**	**Chicken tandoori** Indian dish with marinated chicken, tomato and basmati rice
**490**	**Rosemary chicken** marinated chicken breast with seasoned baby potatoes, haricots verts and rosemary

12. What does this menu labeling format communicates according to you?
(1 = not, 5 = strongly)

Good to prevent heart disease	1	2	3	4	5
Good nutrient composition	1	2	3	4	5
Low energy	1	2	3	4	5
Low in fat	1	2	3	4	5
Conscious choice	1	2	3	4	5
Organic	1	2	3	4	5
Sustainable	1	2	3	4	5
Healthy choice	1	2	3	4	5
Tasteful	1	2	3	4	5

13. To what extent would this menu label format influence restaurant guests' menu choice according to you?
(1= not, 5 =a lot)

	☐ 1 ☐ 2 ☐ 3 ☐ 4 ☐ 5
14.	Which menu label format to communicate that a dish is a healthy and conscious choice do you prefer most?<br>o Logo (menu label 1)<br>o Ranking (menu label 2)<br>o Information (menu label 3)

15.	To what extent do you think implementing menu labeling positively distinguishes you from other restaurants? (1 = not, 5 = a lot)<br>☐ 1 ☐ 2 ☐ 3 ☐ 4 ☐ 5
