# Supplementary material for: What Is on the Menu?—A Quantitative Analysis on Label Format among (Potential) Restaurant Guests and Restaurant Owners
Source: Int J Environ Res Public Health. 2021 Nov 27;18(23):12500. doi: 10.3390/ijerph182312500 (PMC8656631; doi:10.3390/ijerph182312500)
Supplement: Supplementary file 1 [file ijerph-18-12500-s001.zip › Supplementary S2_Table relation guests' characteristics and label preference.pdf]

Supplementary S2

Table S2. Relation between characteristics of the potential guests and label preference (values are represented as numbers (%))

No relation between label preference and the characteristics; gender, monthly income, frequency of visiting a restaurant, reasons to visit an restaurant, health consciousness could be observed. (Pearson's chi-square test goodness of fit).

Characteristics	Logo	Ranking	Information	Total		p-value
**All**	107	164	115	386		
**Gender**						0.184
Female	79	136	89	304	(79%)	
Male	28	28	26	82	(21%)	
**Monthly income (€)**						0.125
<1.500	56	95	62	213	(55%)	
1.500 - < 3.000	37	44	48	129	(33%)	
3.000 - < 5.000	12	10	5	27	(7%)	
5.000 - < 7.000	2	3	1	6	(2%)	
≥ 7.000	0	1	0	1	(0.3%)	
**How often do you visit a restaurant**						0.107
Daily	0	0	1	1	(0.3%)	
Weekly (1 or 2 times per week)	7	11	15	33	(8.5%)	
Monthly (1 or 2 times per month)	42	83	48	173	(44.8%)	
Less than once a month	58	70	51	179	(26.4%)	
**Reason to visit a restaurant**						0.639
Business setting	0	0	1	1	(0.3%)	
Informal setting	87	132	94	313	(81.1%)	
Both business as informal setting	20	32	20	72	(18.7%)	
**Health consciousness***						
Below average	11	11	6	28	(7.3%)	
Average	41	72	41	154	(39.9%)	
Above average	55	81	68	204	(52.8%)	0.184

*Health consciousness was determined based on five questions using a 5-point Likert scale (1=strongly disagree, 5=strongly agree). Outcomes on health consciousness were categorized in three groups, based on the Likert scale (below average (score <2.5), average (score 2.5 – 3.4), above average (score >3.4)). Scores of all five questions were used (Cronbach's alfa = 0.815) to calculate the average participants' health consciousness.
